# Treatment with a sphingosine analog after the inception of house dust mite-induced airway inflammation alleviates key features of experimental asthma

**DOI:** 10.1186/s12931-015-0180-z

**Published:** 2015-02-03

**Authors:** David Gendron, Anne-Marie Lemay, Claudine Tremblay, Laetitia JA Lai, Anick Langlois, Émilie Bernatchez, Nicolas Flamand, Marie-Renée Blanchet, Anthony S Don, Ynuk Bossé, Élyse Bissonnette, David Marsolais

**Affiliations:** Centre de recherche de l’Institut universitaire de cardiologie et de pneumologie de Québec (CRIUCPQ), Québec, QC Canada; Laboratoires Charles River, Services Précliniques, Montréal, Canada; Département de Médecine, Faculté de Médecine, Université Laval, Québec, QC Canada; Prince of Wales Clinical School, Faculty of Medicine, University of New South Wales, Sydney, 2052 NSW Australia

**Keywords:** FTY720, Fingolimod, Gilenya, *Dermatophagoides pteronyssinus*, Apoptosis, Dendritic cells, CD4^+^ T cells, Asthma, S1P, AAL-R, AAL-S, Sphingosine

## Abstract

**Background:**

In vivo phosphorylation of sphingosine analogs with their ensuing binding and activation of their cell-surface sphingosine-1-phosphate receptors is regarded as the main immunomodulatory mechanism of this new class of drugs. Prophylactic treatment with sphingosine analogs interferes with experimental asthma by impeding the migration of dendritic cells to draining lymph nodes. However, whether these drugs can also alleviate allergic airway inflammation after its onset remains to be determined. Herein, we investigated to which extent and by which mechanisms the sphingosine analog AAL-R interferes with key features of asthma in a murine model during ongoing allergic inflammation induced by *Dermatophagoides pteronyssinus*.

**Methods:**

BALB/c mice were exposed to either D. *pteronyssinus* or saline, intranasally, once-daily for 10 consecutive days. Mice were treated intratracheally with either AAL-R, its pre-phosphorylated form AFD-R, or the vehicle before every allergen challenge over the last four days, i.e. after the onset of allergic airway inflammation. On day 11, airway responsiveness to methacholine was measured; inflammatory cells and cytokines were quantified in the airways; and the numbers and/or viability of T cells, B cells and dendritic cells were assessed in the lungs and draining lymph nodes.

**Results:**

AAL-R decreased airway hyperresponsiveness induced by *D. pteronyssinus* by nearly 70%. This was associated with a strong reduction of IL-5 and IL-13 levels in the airways and with a decreased eosinophilic response. Notably, the lung CD4^+^ T cells were almost entirely eliminated by AAL-R, which concurred with enhanced apoptosis/necrosis in that cell population. This inhibition occurred in the absence of dendritic cell number modulation in draining lymph nodes. On the other hand, the pre-phosphorylated form AFD-R, which preferentially acts on cell-surface sphingosine-1-phosphate receptors, was relatively impotent at enhancing cell death, which led to a less efficient control of T cell and eosinophil responses in the lungs.

**Conclusion:**

Airway delivery of the non-phosphorylated sphingosine analog, but not its pre-phosphorylated counterpart, is highly efficient at controlling the local T cell response after the onset of allergic airway inflammation. The mechanism appears to involve local induction of lymphocyte apoptosis/necrosis, while mildly affecting dendritic cell and T cell accumulation in draining lymph nodes.

## Background

Asthma is a chronic lung disorder with no cure that affects 235 million people worldwide [1]. Asthma decreases the quality of life and its yearly cost per country ranges from 10 to 266 billion dollars [2]. Although a myriad of interventions exists to alleviate symptoms, current practices only ensure adequate control of asthma in less than 50% of patients [3]. Furthermore, up to 10 percent of patients suffer from severe forms of asthma that are largely refractory to current therapies [4] and too many still succumb [1]. Given the health and the economic burden of asthma, identification of new mechanisms and targets for intervention is required.

Sphingosine analogs emerge as potent immunomodulators with therapeutic efficacy proven in humans. For instance, the drug FTY720 (Fingolimod, Gilenya) is currently used for patients with multiple sclerosis [5]. The compound AAL-R is an analog of FTY720 that has been broadly used to study the mechanisms of action of this class of compounds [6-9]. Sphingosine analogs such as FTY720 and AAL-R are cell-permeant and become phosphorylated intracellularly to become a sphingosine-1-phosphate (S1P) analog. They are then actively exported from the cells. Owing to their phosphorylation, the secreted forms of the drugs are cell-impermeant and their mechanisms of action become then restricted to extracellular effects, which are mediated by binding on four of the five cell surface S1P receptors (S1P_1,3–5_) [8]. The archetypal mechanism of action of sphingosine analogs is sequestration of lymphocytes in secondary lymphoid organs after activation of S1P_1_ [10]. However, it is increasingly recognized that activation of S1P receptors following the intracellular phosphorylation and secretion of the analogs is not the only mechanism underlying their bioactivity. These compounds can indeed modulate cellular functions independently of S1P receptor engagement, including among others cell fate and the activity of both phospholipase and phosphatase subsets [6,11,12]. The mechanisms of action of sphingosine analogs are thus complex, misunderstood, and might vary under different pathophysiological conditions.

When administered in a prophylactic fashion, phosphorylatable sphingosine analogs interfere with the development of experimental allergic airway inflammation. This was demonstrated in the classical model induced by systemic sensitization with ovalbumin (OVA) emulsified in aluminum (Alum), followed by airway challenges with OVA (OVA-Alum model) [13]. The mechanism of action under these conditions is potent inhibition of dendritic cell (DC) accumulation in draining lymph nodes, interference with antigen presentation, and failure to launch an efficient antigen-specific T cell response [13,14]. However, whether the phosphorylatable sphingosine analogs are salutary when administered in a therapeutic fashion, i.e. after the onset of allergic airway inflammation, is undefined. Additionally, the efficacy of phosphorylatable sphingosine analogs to interfere with airway inflammation induced by a natural allergen has never been studied. In contrast with the classical OVA-Alum model, natural allergens have specific *modi operandi* – including proteolytic activity and endogenous adjuvant activity [15]. Importantly, the natural allergens evoke a classical allergic response by local mechanisms involving pulmonary DC when delivered through natural routes of exposure, such as the airways [16]. They thus recapitulate more genuinely the mechanisms of allergic airway inflammation in humans.

Under these premises, we aimed to determine if a sphingosine analog, namely AAL-R, alleviates experimental asthma elicited by subchronic airway inflammation induced by exposure to a natural allergen, namely *Dermatophagoides pteronyssinus* (house dust mite extract; HDM). We hypothesized that treatment with AAL-R after the onset of HDM-induced airway inflammation interferes with mechanisms leading to key features of asthma in a murine model.

## Methods

### Murine model of asthma

Pathogen-free BALB/c female mice (8 weeks old, Charles River) were anesthetized with isoflurane and instilled intranasally (i.n.) with 50 μl of saline or with saline containing 50 or 75 μg HDM extract (Greer), once daily, from day 1 to 10. Unless otherwise indicated, all measurements were taken at day 11. The mice were either euthanized with ketamine-xylazine overdose for collection of the bronchoalveolar lavage fluid (BALF) and for tissue sampling; or tested for airway responsiveness to methacholine (MCh) using documented methods [17]. Experimental, housing and care procedures were approved by the Committee of Animal Care of Laval University in accordance with the guidelines of the Canadian Council on Animal Care (protocols 2013037, 2009128).

### Immunoglobulin titration

Plates were coated with 50 μg/mL HDM and incubated with serial dilution of serum (1:200 to 1:145800). Titers were determined by a colorimetric reaction as reported [18].

### Experimental treatments

Naïve or HDM-instilled mice were treated intratracheally (i.t.) with either 50 μl of distilled water (dH_2_O) containing 2.5 μg of AAL-R, or the vehicle (dH_2_O) from day 7 to day 10. This dose, which corresponds to 0.1 mg/kg, was chosen because it does not induce histological alterations *per se*, and because it was shown to potently induce immunomodulation in the airways [13,14]. Treatments were delivered under isoflurane anesthesia and animals were thereafter placed in standard housing conditions for 1 h until re-anesthesized with isoflurane prior to HDM instillation. In a second series of experiments, an additional group was treated with a molar equivalent dose (3.5 μg) of the phosphorylated sphingosine analog, namely AFD-R. Compounds were either provided by Dr Hugh Rosen (TSRI) or synthetized using a modification of published methods (NuChem Therapeutics Inc.) [19,20].

### Differential counts, flow cytometric analyses and quantification of cytokines

BALF as well as single-cell suspensions from both the right and central lobes and from draining mediastinal lymph nodes (MLN) were obtained as previously reported [21]. Antibodies are anti- CD4 (RM4-5) (eBioscience); CD8a (53–6.7), CD11c (N418), CD19 (6D5), CD90.2 (30-H12), Ki-67 (16A8) (Biolegend); MHC-II (2G9) (BD Biosciences). Viability was assessed using annexin V (Biolegend) and LIVE/DEAD (Life Technologies). Fluorescence and autofluorescence (AF) were acquired using a Diva-driven LSRFortessa (Becton Dickinson) and analyzed with the FlowJo software (Tree Star inc). To compute the total numbers of cell subsets, absolute cell counts obtained with a hemocytometer were multiplied by cell frequencies obtained either by flow cytometry or following differential cell counts as previously reported [17,21]. For cytokine quantification, cell-free BALF were concentrated with Amicon 3 K (Millipore) and incubated with cytometric bead array Flex Set for interleukin (IL)-5, IL-13 and CCL5 according to the manufacturer’s instructions (BD Biosciences).

### Histology

After BALF were performed, the left caudal lobes were processed as previously described [18] and stained with either hematoxylin and eosin (H&E) for the evaluation of inflammatory cell infiltrate; or with Periodic Acid-Schiff (PAS) stain and hematoxylin for the evaluation of mucus-producing cells. Histological evaluation was performed by an American College of Veterinary Pathologists Board-certified pathologist (CT). The nomenclature used to describe the lesion and the severity of the lesions (5 grades scale) was based on the guidelines provided by the Society of Toxicological Pathology [22,23].

### Measurement of airway responsiveness

Methacholine (MCh) challenge test was performed on tracheotomized mice connected to a flexiVent apparatus (SCIREQ) as previously reported [17].

### Statistical analyses

Unless otherwise stated, the data shown are means ± SEM. Data were analyzed using one-way ANOVAs. To fulfill the normality and variance assumptions, variables were log-transformed. An arc sinus transformation on the square root was applied on BALF measurements that were expressed in percentage. Homogeneity of covariance parameter assumption among groups was verified. *A posteriori* comparisons were performed with Tukey’s comparison technique. All reported *P* values are based on these transformations. In addition, the nonparametric Kruskal-Wallis test was performed on histology data. The results were considered significant with p values ≤ 0.05, unless otherwise stated. All analyses were conducted using the statistical package SAS v9.3 (SAS Institute Inc).

## Results

### Onset of allergic airway inflammation in response to daily exposure to HDM

**Figure 1:**
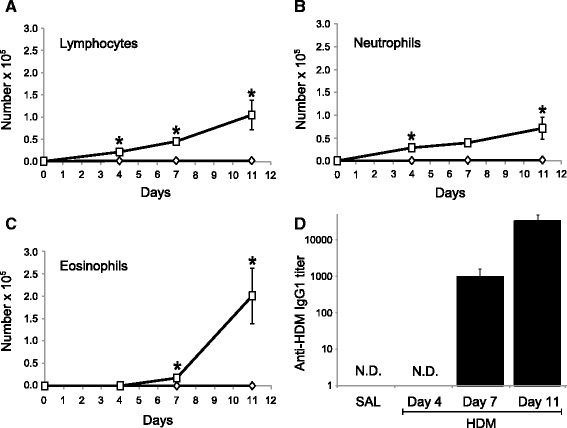
**The onset of allergic inflammation is established at day 7.** Mice received HDM (□) or saline (SAL) (◊) i.n. once-daily for 0, 3, 6 or 10 days and were then euthanized 24 h after the last exposure. BALF were performed and differential cell counts were determined for **A)** lymphocytes, **B)** neutrophils, and **C)** eosinophils. **D)** Serum HDM-specific IgG1 titers were also quantified. n = 4 per group and *indicates significant differences in corresponding days between SAL- and HDM-exposed mice. *P* <0.05 N.D.: not detected.

**Figure 2:**
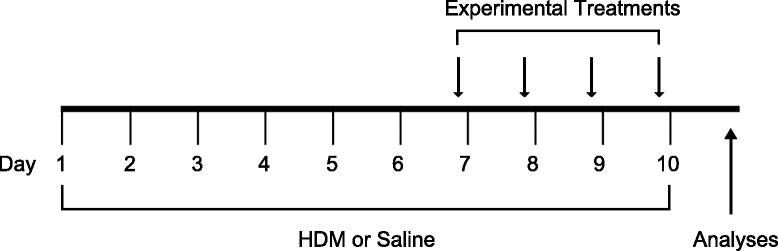
**Experimental design.** Mice were instilled i.n. with HDM or saline (SAL) from day 1 to 10, along which time they were also treated i.t. with vehicle (dH_2_O) or dH_2_O containing 2.5 μg of AAL-R from day 7 to day 10, one hour before HDM administration. In a subset of experiments, 3.5 μg of AFD-R (molar equivalent of AAL-R) were used instead of AAL-R. 24 h after the last exposure, mice were either euthanized for the collection of BALF and tissue sampling or tested to assess their degree of airway responsiveness to MCh.

In order to ensure that our experimental treatments were undertaken after the inception of local allergic inflammation, we assessed the kinetics of the systemic and the local allergic responses in this subchronic model induced by HDM. Inflammation was evaluated in the BALF at day 4, 7, and 11. Daily i.n. delivery of saline failed to induce accumulation of leukocytes in the BALF (Figure 1). In contrast, HDM delivery was associated with a mild increase of absolute numbers of lymphocytes (0.22 ± 0.02 × 10^5^, Figure 1A) and neutrophils (0.29 ± 0.08 × 10^5^, Figure 1B) in BALF on day 4. Instillation of HDM also induced the recruitment of eosinophils in the airways with 0.18 ± 0.07 × 10^5^ eosinophils in BALF at day 7 (Figure 1C). The number of eosinophils reached 2.0 ± 0.6 × 10^5^ at day 11. The appearance of antigen-specific IgG1 (functional equivalent to human IgE) in serum (Figure 1D) coincided with the onset of eosinophilia, validating that sensitization had occurred at that time-point. These results indicated that the onset of allergic airway inflammation was clearly established at day 7 and it was thus a proper time for initiating the treatment with sphingosine analogs (Figure 2).

### AAL-R alleviates airway responsiveness induced by HDM

**Figure 3:**
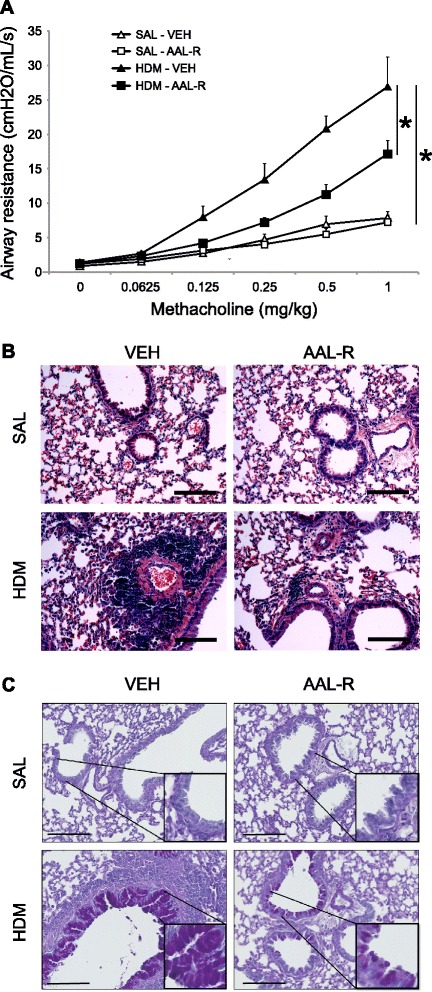
**AAL-R interferes with hallmarks of asthma induced by HDM. A)** The degree of airway responsiveness was assessed by monitoring the change of respiratory system resistance (Rrs) during doubling doses of MCh. Paraffin-embedded tissue sections of lung stained with either H&E to assess **B)** inflammatory infiltrates in the peribronchial/perivascular regions or **C)** PAS to label mucus-producing cells. n = 4–5 mice per group and *indicates significant differences *P* <0.05. bar = 150 μm.

In patients with allergic asthma, the sensitization phase has already occurred and exacerbations arise due to inflammatory flares triggered by allergen exposures. To be physiologically meaningful, a treatment may thus be able to alleviate pathognomonic features of asthma after its onset. We first determined whether local delivery of AAL-R after the onset of allergic airway inflammation interferes with alteration of respiratory function (Figure 3A). Mice treated daily with saline showed a weak increase of respiratory system resistance (Rrs) in response to MCh with a delta of 7.1 ± 1.1 cmH_2_O/mL/s between baseline (0.4 cmH_2_O/mL/s) and the 1 mg/kg dose. Mice receiving HDM showed a dose-dependent increase of Rrs that reached 26.9 ± 4.3 cmH_2_O/mL/s at the highest MCh dose. In saline-treated mice, AAL-R did not modulate Rrs in response to MCh. On the other hand, AAL-R inhibited HDM-induced airway hyperresponsiveness by as much as 70% at doses of 0.25 and 0.5 mg/kg of MCh. The functional advantage provided by AAL-R was associated with amelioration of histological features. For instance, AAL-R decreased median perivascular and peribronchial accumulation of mononuclear cells (Figure 3B; Table 1) as well as HDM-associated alteration of the epithelium, including PAS reactivity and epithelial layer hypertrophy (Figure 3C, Table 1). Thus, treatment with AAL-R inhibits functional and histological alterations caused by HDM instillation.

**Table 1:** **Histopathological alterations of mice lungs exposed to HDM**

	**Saline**		**HDM**	
**Treatments**	**VEH**	**AAL-R**	**VEH**	**AAL-R**
Perivascular infiltration of mononuclear cells	0	0	2^*^	1^†^
Peribronchial/bronchiolar infiltration of mononuclear cells	0	0	2^*^	1^†^
Perivascular infiltration of granulocytes	0	0	2^*^	1
Hypertrophy/hyperplasia of the bronchial epithelium	0	0	3^*^	1^†^

Results are expressed as median scores for each group, graded on a scale from 0 (no alteration) to 5 (severe alterations).

4–5 mice per group were analyzed. ^*^ *P* < 0.001 between HDM and Saline; ^†^ *P* < 0.01 between AAL-R and vehicle.

### AAL-R interferes with lung accumulation of cells that drive inflammation induced by HDM

**Figure 4:**
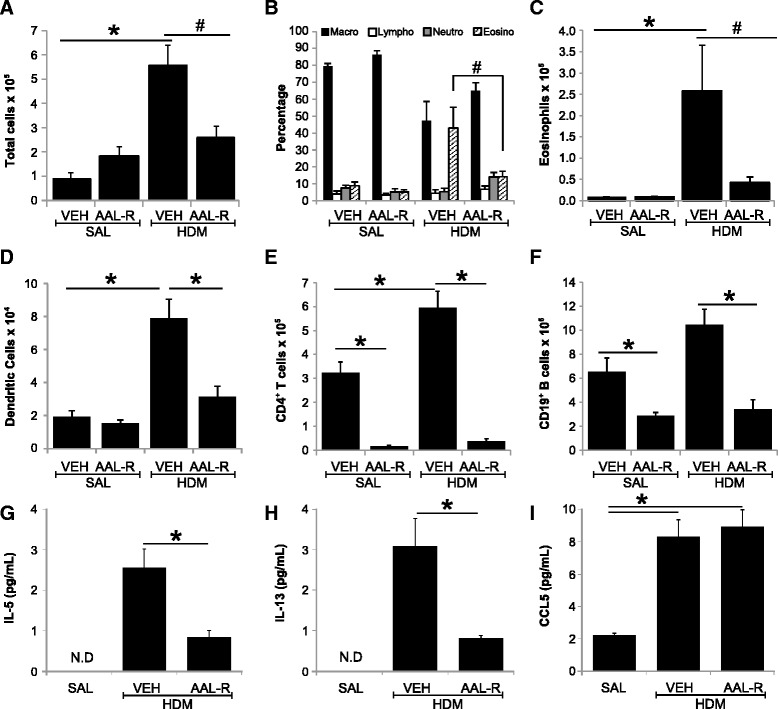
**AAL-R inhibits allergic airway inflammation.** BALF was assessed to determine: **A)** the total number of cells; **B)** the differential count of cells; and **C)** the total number of eosinophils. Inflammation was also assessed in single-cell suspensions derived from digested lungs to determine: **D)** The total number of AF^neg^ MHC-II^hi^ CD11c^hi^ DC; **E)** the number of lung AF^neg^ SSC^low^ CD90.2^+^ CD8^neg^ CD4^+^ T cells; and **F)** the number of lung AF^neg^ SSC^low^ CD90.2^neg^ CD19^+^ B cells. Levels of **G)** IL-5, **H)** IL-13, and **I)** CCL5 were also quantified in BALF. Shown is representative of two independent experiments. n = 4–5 per group and *signifies significant differences at *P* <0.05 (^#^P = 0.07).

We then set out to investigate whether and by which mechanisms AAL-R affects the ongoing inflammatory response to HDM in the lungs. The effect of AAL-R on the leukocyte profile in the BALF was first investigated (Figure 4). AAL-R had no drastic effect on numbers of leukocytes in the group receiving saline instead of HDM. The absolute number of leukocytes decreased from 5.6 ± 0.8 × 10^5^ in vehicle-treated HDM mice to 2.6 ± 0.4 × 10^5^ in AAL-R-treated HDM mice (Figure 4A). In mice receiving saline, AAL-R did not affect the proportion of different cell subsets in the BALF (Figure 4B). No eosinophilia was detected in the saline groups and the percentage of eosinophils was increased to 43 ± 12 by HDM, which was reduced to 14 ± 3 with AAL-R (Figure 4B). This resulted in an absolute decrease of eosinophil number in the BALF (Figure 4C). In cell suspensions prepared from the lungs, AAL-R decreased AF^neg^ CD11c^hi^ MHCII^hi^ DC numbers by more than 50% in mice exposed to HDM (Figure 4D), which was accompanied by a 90% decrease of pulmonary CD4^+^ T cells (Figure 4E) and by a 70% decrease of pulmonary CD19^+^ B cells (Figure 4F), when compared with the vehicle-treated HDM-exposed group. The decrease in lymphocyte numbers in AAL-R-treated mice also occurs in conjunction with a decrease in lymphocyte-secreted cytokines IL-5 and IL-13 (Figure 4G-H), while having no effect on CCL5, a chemokine predominantly secreted by epithelial cells (Figure 4I). These results support the efficacy of locally-delivered AAL-R to interfere with accumulation of cells critically involved in the immunopathological responses seen in allergic asthma.

### AAL-R enhances apoptosis/necrosis of lymphocytes in the lung

**Figure 5:**
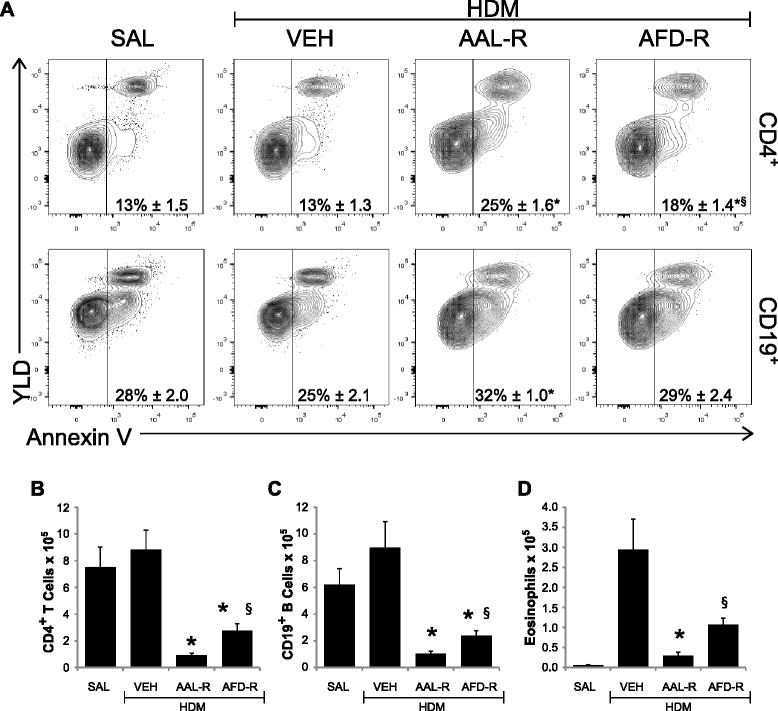
**AAL-R increases lymphocyte apoptosis/necrosis in the lungs.** Single-cell suspensions were obtained from the lungs to determine: **A)** the frequency of apoptotic/necrotic annexin V^+^ cells in two subpopulations of cells, namely AF^neg^ SSC^low^ CD90.2^+^ CD8^neg^ CD4^+^ T cells (upper panels) and AF^neg^ SSC^low^ CD90.2^neg^ CD19^+^ B cells (lower panels); **B)** the number of CD4^+^ T cells; and **C)** the number of CD19^+^ B cells in the lung. Total number of eosinophils was also determined in the BALF **(D)**. Shown is representative of two independent experiments. n = 6–8 mice per group and *indicates significant differences versus VEH; ^§^indicates significant differences versus AAL-R. *P* <0.05.

Although a rich literature supports sphingosine analogs to induce apoptosis, whether or not this mechanism is involved in the decrease of leukocyte numbers in the lungs remains undetermined. As previously reported [24] apoptotic/necrotic lymphocytes were detected in the lungs under basal conditions (Figure 5). The percentage of annexin V^+^ CD4^+^ T cells changed from 13.3 ± 1.3 in vehicle-treated HDM-exposed mice to 24.6 ± 1.6 in HDM-exposed mice treated with AAL-R (Figure 5A). Similarly, the proportion of CD19^+^ B cells positive for annexin V increased by 27.9% in AAL-R-treated mice (Figure 5A). Weak to null apoptosis/necrosis was induced in both CD8^+^ T cells and the CD90^−^CD19^−^AF^−^SSC^med-hi^ granulocyte-enriched fraction (not shown), supporting cell type-specific induction of cell death in the lungs. In contrast, a molar equivalent dose of the phosphorylated AAL-R analog, AFD-R, failed to induce potent apoptosis/necrosis of CD4^+^ T cells and CD19^+^ B cells, when compared with the vehicle-treated HDM-exposed group. Notably, AFD-R was less efficient than AAL-R to interfere with the T cell and B cell responses in the airways (Figure 5B-C), when compared with AAL-R. Accordingly, AFD-R was also less efficient than AAL-R to inhibit the eosinophilic response (Figure 5D). Thus, a phosphorylatable sphingosine analog, but not its pre-phosphorylated form, potently induces death of cell subsets that are central to allergic airway inflammation.

### Effect of intratracheal delivery of AAL-R on lymphocytes and DC in the draining lymph nodes

**Figure 6:**
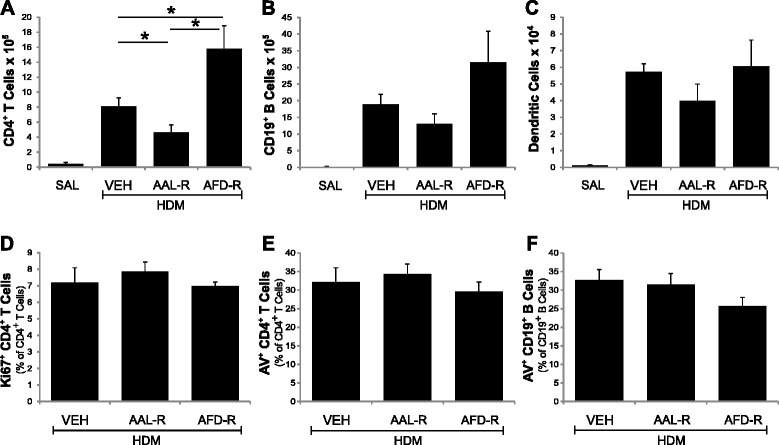
**Effects of non- or pre-phosphorylated analogs in draining lymph nodes.** Single-cell suspensions derived from MLNs were obtained to determine: **A)** the total number of AF^neg^ SSC^low^ CD90.2^+^ CD8^neg^ CD4^+^ T cells; **B)** the total number of AF^neg^ SSC^low^ CD90.2^neg^ CD19^+^ B cells; **C)** the total number of MHC-II^hi^ CD11c^hi^ DC; **D)** the frequency of Ki67^+^ CD4^+^ T cells; **E)** the frequency of apoptotic/necrotic CD4^+^ T cells; and **F)** the frequency of apoptotic/necrotic CD19^+^ B cells. Apoptosis and proliferation could not be evaluated in saline mice given the paucity of cells. n = 6–8 mice per group and *signifies significant differences *P* <0.05. For panels A to C, means of all experimental groups are statistically different from SAL. Shown is representative of two independent experiments.

We next addressed the ability of AAL-R delivered into the lungs to interfere with the biology of draining MLNs. Compared with vehicle-treated mice with experimental asthma, AAL-R mildly reduced CD4^+^ T lymphocyte numbers in the MLNs (Figure 6A) and failed to modulate CD19^+^ B cell and DC numbers in this compartment (Figure 6B-C). This is in agreement with the lack of effect on the frequency of proliferative Ki67^+^ CD4^+^ T cells (Figure 6D). AAL-R also failed to modulate the percentage of annexin V^+^ CD4^+^ cells and annexin V^+^ CD19^+^ cells in MLNs (Figure 6E-F). In contrast, AFD-R increased the numbers of CD4^+^ T cells in MLNs by 85 %; albeit equally ineffective as AAL-R to alter DC numbers. Thus, on one hand, it appears that AAL-R acts directly in the lungs without affecting leukocyte numbers in the draining lymph nodes. On the other hand, the pre-phosphorylated form (AFD-R) seems to preferentially attenuate cell numbers in the lungs by sequestering lymphocytes in the draining lymph nodes.

## Discussion

Sphingosine analogs are new therapeutic molecules with potent immunomodulatory properties. Yet, their potential in the treatment of asthma is misunderstood. The main finding of this study is the efficacy of a phosphorylatable sphingosine analog to interfere with asthma in a murine model after the onset of allergic inflammation induced by a natural allergen. We also reveal that lymphocyte and DC numbers in draining lymph nodes are not modulated by AAL-R, as it was previously observed in the OVA-Alum model of asthma [14]. Conversely, AAL-R attenuates the accumulation of eosinophils, DC, CD4^+^ T cells and CD19^+^ B cells in the lungs. This was associated with significant decreases of IL-5 and IL-13 levels in the BALF. We also identify the induction of T cell apoptosis as a significant mechanism contributing to the alleviation of allergic airway inflammation by AAL-R; and this phenomenon was not observed using the pre-phosphorylated cell impermeant form of the drug.

Previous studies have addressed the roles of S1P pathway modifiers in the phase of intra peritoneal sensitization with OVA-Alum [25]. In addition, administration of S1P pathway modifiers prior to, or concomitantly with, repetitive OVA challenges in peripherally sensitized animals was shown to be effective in preventing cardinal features of experimental asthma [14]. However, it was heretofore unknown whether the drugs were able to attenuate key features of established allergic airway inflammation. The findings of the present study represent the first evidence that a phosphorylatable sphingosine analog can alleviate experimental asthma after the onset of allergic airway inflammation.

It is becoming clear that the airway mucosa can be a critical site for amplification and maintenance of allergic disease [26]. Consequently, models using natural allergens that cause signs of experimental asthma when delivered through natural routes are currently preferred to assess the preclinical potential of experimental treatments [27]. In the model used herein, airway delivery of HDM was sufficient to trigger functional impairment of the airways, accumulation of T cells, B cells and eosinophils. Remarkably, all of which were inhibited by AAL-R. In accordance with our current finding, FTY720 dampens eosinophil accumulation within the mucosa in a model of ongoing allergic rhinitis [28]. Unfortunately, the number of DC in draining lymphoid tissues was not assessed in that latter study [28]. In our study, both local DC and T cells were severely altered by local treatment with AAL-R, which is a major interest owing to the predominant role played by these cell subsets in pulmonary inflammation. This alteration of the local T cell response was also associated with a strong decrease of IL-5 and IL-13 levels in the airways. Of potential importance is the fact that these interleukins can by themselves increase the contractile capacity of airway smooth muscle [29]. It is thus likely that AAL-R interfered with airway hyperresponsiveness, at least in part, by reducing the levels of soluble factors that potentiate the contractile capacity of airway smooth muscle. On note, airway smooth muscle enlargement is not observed in this acute model; so impairment of this feature may not be related to the beneficial effect of AAL-R on airway hyperresponsiveness. The location of T cells has also recently emerged as a critical mechanism influencing the contractile capacity of airway smooth muscle. Indeed, T cells in the vicinity of airway muscle cells modify their contractile capacity [30,31]. It is tempting to speculate that the profound reduction of T cell numbers by AAL-R might have contributed to alleviate hyperresponsiveness by reducing the number of T cells located proximally to the contractile apparatus of the airways.

The current work also highlights that the immunomodulatory mechanisms of sphingosine analogs after the onset of allergic inflammation likely differ from the ones observed in the context of a prophylactic treatment. When administered prophylactically, i.e. prior to airway OVA challenge, sphingosine analogs strongly inhibit antigen presentation in draining lymph nodes, which is responsible for the alleviation of experimental allergic airway disease [13,14]. In agreement with these results, airway delivery of a single dose of AAL-R soon after influenza virus infection inhibits both DC accumulation in draining lymph nodes and the ensuing immunopathological responses in the airways [9,32]. Conversely, our results show that AAL-R strongly inhibits DC accumulation in the lungs while having mild effects on DC numbers in MLNs over the course of a 4-day treatment. These results are in line with the ability of sphingosine analogs to interfere with *de novo* DC recruitment from blood to tissues [33], and to blunt the release of chemotactic factors in the lung [9,13]. While we cannot fully exclude the possibility that antigen presentation was modulated, our results rather suggest that once DCs have migrated to the lymph node, sphingosine analogs are relatively impotent at interfering with the chain of events leading to the antigen-triggered amplification of lymphocytes.

Although several studies used local delivery of sphingosine analogs [9,13,14,21,34,35], and sphingosine analogs are well known inducers of cell death [11], this is the first evidence supporting the idea that cell death could contribute to alleviate allergic airway disease. Indeed, aminoalcohols like AAL-R readily penetrate the plasma membrane and become phosphorylated intracellularly to interfere with cell survival [11]. Accordingly, we show that AAL-R, but not AFD-R, potently induces lymphocyte apoptosis/necrosis in the lung. This is consistent with AAL-R’s rapid phosphorylation, low turnover, and potent induction of apoptosis in murine splenocytes through intracellular accumulation of AFD-R [7]. Interestingly, AAL-R-induced apoptosis/necrosis appears to affect CD4^+^ T cells and B cells, which are crucially involved in allergic airway disease, while having no effect on CD8^+^ T cells, DCs and the CD90^−^CD19^−^AF^−^SSC^med-hi^ granulocyte-enriched fraction. These results certainly argue against a non-specific toxic effect. Of note, FTY720 was associated with preferential apoptosis of activated T cells in another model [36], while not potently affecting DC apoptosis *in vitro* [37] and myeloid leukocyte apoptosis *in vivo* [38]. In a preclinical perspective, drugs that are customarily used to treat asthma can induce apoptosis in cells from the airways, supporting the viability of this drug-related mechanism of action in humans [39,40].

Lymphopenia has long been associated with the immunomodulatory effects of sphingosine analogs. Yet, a number of facts challenge the idea that systemic lymphopenia *per se* is central to immunomodulation. Indeed, local treatments with sphingosine analogs induce more potent immunosuppression than systemic delivery [9]. In addition, naïve T cells are more sensitive to sphingosine analogs-induced lymphopenia than activated T cells [41]. Our results reveal that distinct mechanisms of action could be associated with the non-phosphorylated and the phosphorylated forms of the compounds. Specifically, our results demonstrated that AAL-R induces a more severe local T cell inhibition than AFD-R, which correlates with the ability of AAL-R to induce local death of lymphocytes. The idea that lymphopenia-independent mechanisms could explain, at least in part, the local immunosuppression induced by sphingosine analogs is also supported by the observation that prophylactic treatment with the non-phosphorylatable AAL-S enantiomer, yet at a somewhat superior dosage than in the current study, inhibits cytokine release as well as T cell and DC accumulation in the airways in a model of experimental asthma [6]. This effect was associated with activation of protein phosphatase 2A, but it remains unknown if such mechanism is viable under the current conditions.

## Conclusion

We conclude that a phosphorylatable sphingosine analog can interfere with allergic airway inflammation caused by a natural allergen, resulting in improved respiratory function. The mechanism of action appears to differ from that previously observed in a prophylactic context. Our results also support that the phosphorylation status of the drug can influence the mechanisms by which the immune response is modulated, with enhancement of T cell apoptosis being restricted to the non-phosphorylated form.
